# Alterations of lipid metabolism provide serologic biomarkers for the detection of asymptomatic versus symptomatic COVID-19 patients

**DOI:** 10.1038/s41598-021-93857-7

**Published:** 2021-07-09

**Authors:** Alhaji H. Janneh, Mohamed Faisal Kassir, Connor J. Dwyer, Paramita Chakraborty, Jason S. Pierce, Patrick A. Flume, Hong Li, Satish N. Nadig, Shikhar Mehrotra, Besim Ogretmen

**Affiliations:** 1 Department of Biochemistry and Molecular Biology, Medical University of South Carolina, 86 Jonathan Lucas Street, Charleston, SC 29425 USA; 2 Hollings Cancer Center, Medical University of South Carolina, 86 Jonathan Lucas Street, Charleston, SC 29425 USA; 3 Department of Surgery, Medical University of South Carolina, 86 Jonathan Lucas Street, Charleston, SC 29425 USA; 4 Department of Medicine, Medical University of South Carolina, 86 Jonathan Lucas Street, Charleston, SC 29425 USA; 5 Department of Public Health Sciences, Medical University of South Carolina, 86 Jonathan Lucas Street, Charleston, SC 29425 USA

**Keywords:** Biochemistry, Lipidomics, Biomarkers, Predictive markers

## Abstract

COVID-19 pandemic exerts a health care emergency around the world. The illness severity is heterogeneous. It is mostly unknown why some individuals who are positive for SARS-CoV-2 antibodies stay asymptomatic while others show moderate to severe disease symptoms. Reliable biomarkers for early detection of the disease are urgently needed to attenuate the virus’s spread and help make early treatment decisions. Bioactive sphingolipids play a crucial role in the regulation of viral infections and pro-inflammatory responses involved in the severity of COVID-19. However, any roles of sphingolipids in COVID-19 development or detection remain unknown. In this study, lipidomics measurement of serum sphingolipids demonstrated that reduced sphingosine levels are highly associated with the development of symptomatic COVID-19 in the majority (99.24%) SARS-CoV-2-infected patients compared to asymptomatic counterparts. The majority of asymptomatic individuals (73%) exhibited increased acid ceramidase (AC) in their serum, measured by Western blotting, consistent with elevated sphingosine levels compared to SARS-CoV-2 antibody negative controls. AC protein was also reduced in almost all of the symptomatic patients’ serum, linked to reduced sphingosine levels, measured in longitudinal acute or convalescent COVID-19 samples. Thus, reduced sphingosine levels provide a sensitive and selective serologic biomarker for the early identification of asymptomatic versus symptomatic COVID-19 patients.

## Introduction

The Coronavirus disease 2019, COVID-19, pandemic is an urgent national health emergency due to the outbreak of the novel coronavirus (SARS-CoV-2) infection that continues spreading at dangerous levels with a high level of morbidity in the United States and around the world. Recent studies suggest that mortality due to COVID-19 is mainly attributed to the hyperinflammatory response leading to cytokine storm and acute respiratory distress syndrome (ARDS) in infected patients^1–3^. It is known that in severe cases of COVID-19, patients showed increased serum cytokine levels of IL-2, TNFα, IL-1β, IFNγ, MCP-1, MIP1α, IL-10, and IL-6. ARDS was reported to be developed more often in elderly COVID-19 patients due to a cytokine storm^4–8^.

The severity of COVID-19 disease and the infection course is heterogeneous and appears to be more severe in the elderly and individuals with underlying comorbidities, including cancer^9,10^, pregnant women^11^, hypertension^12–14^, and obesity^15^. However, modifiers of the immune response and their mechanisms remain unknown. We also do not know why some patients develop severe COVID-19, while others stay asymptomatic. Thus, while the infected patient’s overall immunity cannot explain this broad spectrum in disease presentation, differences in immune responses and metabolic alterations might be involved in the disease’s progression to severe stages. Although successful vaccines against SARS-CoV-2 are now in place for disease prevention, early diagnosis, and isolation of mild or asymptomatic COVID-19 patients using selective biomarkers is essential to control the spread of SARS-CoV-2. However, the clinical characteristics of these asymptomatic individuals remain elusive.

The differences in specific immune responses required to eliminate the virus and prevent disease progression to severe stages might be involved. For example, it has been shown that after the SARS-CoV-2 infection, CD4^+^T lymphocytes are rapidly activated to become pathogenic T helper (Th) 1 cells and generate GM-CSF, which then induces inflammatory CD14^+^CD16^+^ monocytes with high expression of IL-6 and accelerate the inflammation^16,17^. These inflammatory monocytes then enter the pulmonary circulation in huge numbers and play a damaging immune role, causing lung functional disability and quick mortality. The cytokine release syndrome (CRS) was reported to affect patients with severe conditions^8^. While effector T cells exhibit increased aerobic glycolysis^18^, memory T cells utilize oxidative phosphorylation (OXPHOS)^19^. Furthermore, molecules such as AMPK^20^, HIF1α^21^, and Foxo1^22,23^ dictate the balance between effector and memory T cells. The dependence of memory T cells on fatty acid oxidation and lysosomal lipolysis^24^ has been shown. In addition to mitochondrial biogenesis, the mitochondria’s quality, as observed by the cristae organization, also influences T cell fitness and its ability to control tumors and viral infections^25^. Recently, Th17 cells gained increased attention because their “stem cell-like” characteristics enable them to persist longer in the host^18,26^. Increased effector T cell function controls tumors and exhibits superior anti-viral activity to control SARS-CoV-2/COVID-19 is mainly associated with increased glutaminolysis and increased dependence on mitochondrial metabolism^27^. Thus, determining metabolic modifiers, including lipid metabolism, will likely help define the mechanisms of immune responses elicited by SARS-CoV-2 viral infection.

Sphingolipid metabolism is known to regulate inflammation and immune response. Previous studies demonstrated that sphingolipid metabolism, through the conversion of sphingosine to sphingosine 1-phosphate (S1P), induces immune response and inflammation by creating an S1P gradient, resulting in the egress of lymphocytes from lymphoid organs to the bloodstream^28,29^. S1P analogs or antagonists that inhibit inflammatory signaling in *Mycobacterium tuberculosis* (H37Ra) infected macrophages prevented pro-inflammatory response and resulted in the clearance of bacterial load^30^. Additionally, S1P signaling was identified as a potential target to play essential roles in pulmonary disorder in response to H1N1 influenza virus infection in mice with decreased IFNγ, TNFα, CCL2, CCL5, CXCL10, IL-1rα, and IL-6 secretion^31,32^. Mechanistically, while S1P induces PI3K/AKT and NFκB activation, sphingosine plays opposing roles and inhibits pro-inflammatory cytokine release via mostly dephosphorylation of AKT and p65 subunit of NFκB^33^. Recently, FTY720 (Fingolimod, Novartis), a sphingosine analog and immune modulator drug was introduced in the clinical trial to determine its role as an immunomodulator in COVID-19 patients^34,35^. Interestingly, a recent study demonstrated that sphingosine inhibited Spike protein’s interaction with ACE receptors, suggesting its potential role in preventing SARS-CoV-2 viral infection^36^. Sphingosine can be generated by the metabolism of ceramide with the function of ceramidases, including acid ceramidase (AC, encoded by ASAH1 gene) known to be secreted to the serum in its active form^37,38^. However, the clinical significance of sphingolipid alterations concerning the COVID-19 disease outcomes has not been reported previously.

Thus, in this study, we investigate the potential clinical utility of serologic sphingolipid levels measured using mass spectrometry-based lipidomics. Our goal is early detection of individuals who are positive for an antibody that recognizes SARS-CoV-2-specific Spike protein to exhibit asymptomatic versus symptomatic disease outcomes based on their serologic sphingosine (lipid) and active form of acid ceramidase (AC protein) levels.

## Results

### Reduced serum sphingosine is associated with symptomatic COVID-19 patients compared to asymptomatic donors who are SARS-CoV-2 antibody-positive

**Figure 1:**
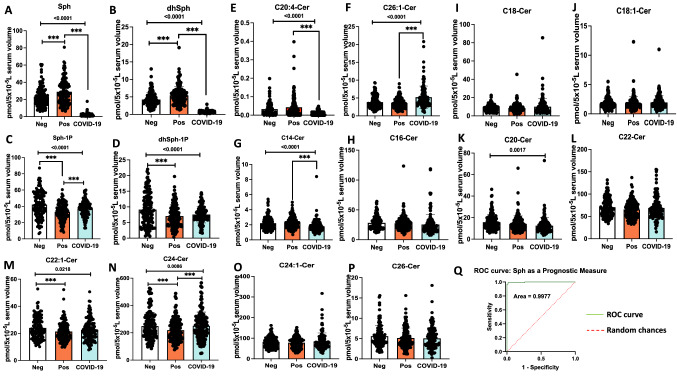
Decreased serum sphingosine level is linked with COVID-19 symptoms. Lipidomics measurements based on High-Performance Liquid Chromatography-tandem Mass Spectrometry (LC–MS/MS) for (**A**) Sphingosine (Sph), (**B**) dihydro-sphingosine (dhSph), (**C**) sphingosine 1-phosphate (Sph-1p), and (**D**) dihydro-sphingosine 1-phosphate (dhSph-1p). LC–MS/MS ceramide (Cer) measurements for (**E**) C20:4-Cer, and (**F**) C26:1 Cer. Moreover, LC–MS/MS ceramide (Cer) measurements for (**G**) C14-Cer, (**H**) C16-Cer, (**I**) C18-Cer, (**J**) C18:1-Cer, (**K**) C20-Cer, (**L**) C22-Cer, (**M**) C22:1-Cer, (**N**) C24-Cer, (**O**) C24:1-Cer, and (**P**) C26-Cer. (**Q**) Graphical ROC (receiving-operating characteristics) curve for Sph as a prognostic measure, produced by plotting Sensitivity(true positive rate) against 1-Specificity (false positive rate). Area under the ROC curve is 0.9977 or 99.77%. The light green line (ROC curve) shows combinations of sensitivity and specificity for threshold values, while the red dotted line (Random chances) indicates a reference cut-off point for a useful model. *Neg* negative antibody test (n = 130), *Pos* asymptomatic positive antibody test (n = 134), *COVID-19* symptomatic patients (n = 131). Data are means of ± SEM, and P < 0.05 is considered significant. Comparisons significant at the 0.05 level are indicated by ***.

To assess whether there are any alterations in sphingolipid metabolism in asymptomatic individuals who are serologically positive versus negative for the SARS-CoV-2 antibody, we measured ceramides, sphingosine, and S1P levels in their serum samples using quantitative high performance liquid chromatography-tandem mass spectrometry (HPLC–MS/MS)-based lipidomics approach. The data showed that there is a slight but significant increase in the levels of sphingosine (p < 0.05) in individuals who are antibody positive (n = 134) compared to negative (n = 130), with sphingosine levels 28.96 versus 23.25 pmol/5 × 10^−5^ L serum, respectively (Fig. 1A). We then determined whether serum sphingosine levels also altered in individuals who were serologically positive for the SARS-CoV-2 antibody and clinically symptomatic (n = 131) for COVID-19 versus asymptomatic donors. The data showed that COVID-19 patients’ serum sphingosine levels were around 15-fold decreased compared to that of asymptomatic donors from 28.96 to 1.88 pmol/5 × 10^−5^ L serum, respectively (Fig. 1A). These results were also consistent with an around sevenfold decrease in dihydro(dh)-sphingosine levels in serum samples obtained from COVID-19 patients compared to asymptomatic donors with 0.74 and 5.4 pmol/5 × 10^−5^ L serum, respectively (Fig. 1B). Decreased serum sphingosine in COVID-19 patients compared to asymptomatic donors was not associated with S1P levels in serum (Fig. 1C,D), which were comparable, suggesting that metabolism of S1P might not play a role in regulating sphingosine levels in COVID-19 patients or asymptomatic donors.

Sphingosine can be generated in response to ceramide's hydrolysis by ceramidases, including acid ceramidase (AC, encoded by ASAH1 gene), which is known to be secreted in its active form to regulate blood levels of sphingosine in various models. Thus, we also measured ceramides with different fatty acyl chain lengths, including short, long, and very-long-chain fatty acyl chains (C14/16-, C18/C20-, and C22/C26-ceramides, respectively). Interestingly, while there were no considerable changes in short-chain ceramides, the levels of C20:4-ceramide were decreased, and C26:1-ceramide were increased (Fig. 1E–P) in COVID-19 patients compared to asymptomatic donors. These data suggest that the metabolic balance between ceramide and sphingosine might play a role in altered serum sphingosine levels in COVID-19 patients or asymptomatic donors. Additionally, our analysis of the ROC (receiving-operating characteristics) curve for reduced sphingosine levels in COVID-19 patients gave rise to 99.77% area under the curve (Fig. 1Q). From the ROC analysis, a sphingosine threshold (or cut-off) value of 8.2 pmol/5 × 10^−5^ L resulted in 98.47% (95% CI 94.60–99.73%) sensitivity and 98.51% (95% CI 94.72–99.73%) specificity, suggesting that serum sphingosine level provides a selective and sensitive biomarker to identify symptomatic patients versus asymptomatic donors who are positive for SARS-CoV-2 antibody.

**Figure 2:**
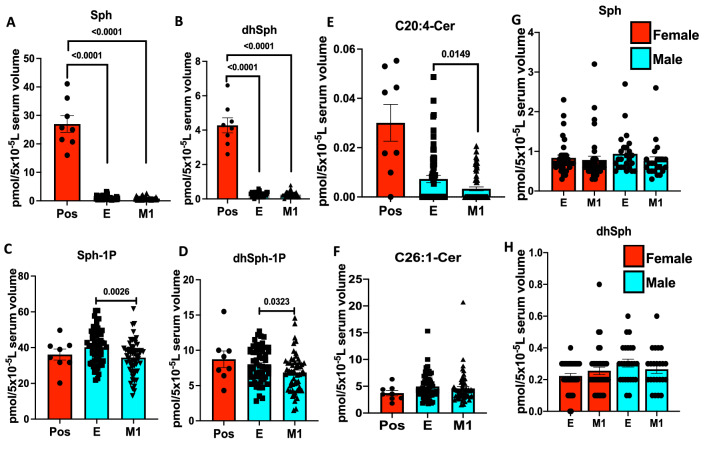
Reduced serum sphingosine level in COVID-19 patients, is not associated with the diseases’ severity. LC–MS/MS measurements for (**A**) Sphingosine (Sph), (**B**) dihydro-sphingosine (dhSph), (**C**) sphingosine 1-phosphate (Sph-1p), and (**D**) dihydro-sphingosine 1-phosphate (dhSph-1p). LC–MS/MS ceramide (Cer) measurements for (**E**) C20:4-Cer, and (**F**) C26:1 Cer. (**G**,**H**) Sph (**G**) and dhSph (**H**) levels of symptomatic COVID-19 patients in male and female patients. *Pos* asymptomatic positive antibody test (n = 8), *E* enrolled at time of diagnosis (n = 60), *M1* one month of recovery (n = 59). Data are means of ± SEM, and P < 0.05 is considered significant.

**Figure 3:**
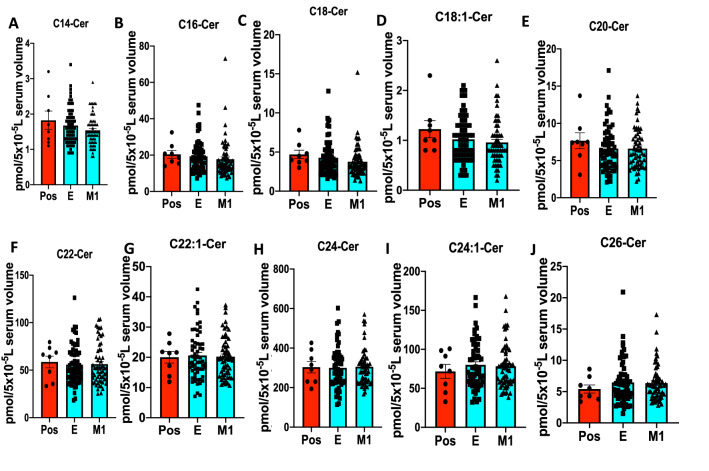
Serum ceramide levels with different fatty acyl chain lengths in COVID-19 longitudinal samples compared to asymptomatic positives. LC–MS/MS ceramide (Cer) measurements for (**A**) C14-Cer, (**B**) C16-Cer, (**C**) C18-Cer, (**D**) C18:1-Cer, (**E**) C20-Cer, (**F**) C22-Cer, (**G**) C22:1-Cer, (**H**) C24-Cer, (**I**) C24:1-Cer, and (**J**) C26-Cer. *Pos* Asymptomatic Positive antibody test (n = 8), *E* enrolled at time of diagnosis (n = 60), *M1* one month of recovery (n = 59). Data are means of ± SEM, and P < 0.05 is considered significant.

**Figure 4:**
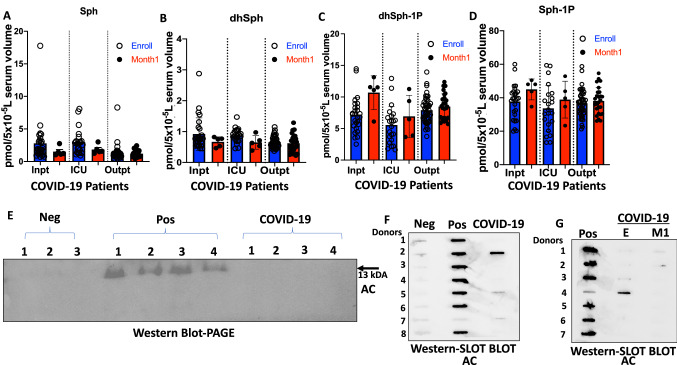
Elevated acid ceramidase level in asymptomatic serum is positively correlated with sphingosine levels. LC–MS/MS measurements for (**A**) sphingosine (Sph), (**B**) dihydro-sphingosine (dhSph), (**C**) dihydro-sphingosine 1-phosphate (dhSph-1p), and (**D**) sphingosine 1-phosphate (Sph-1p). (**E**) Representative western blot image detecting AC (mature alpha-subunit) in equal volumes (24 µl) of serum for Neg (n = 18), Pos (n = 22), and COVID-19 (n = 22). Representative slot blot images detecting AC in equal volumes (15 µl) of serum for (**F**) Neg (n = 23), Pos (n = 114), and COVID-19 (n = 23), and (**G**) Pos (n = 59), E (n = 59), and M1 (n = 59). *Inpt* inpatient (E, n = 27; M1, n = 5), *ICU* Intensive Care Unit (E, n = 22; M1, n = 5); *Outpt* outpatient (E, n = 46; M1, n = 26). *Pos* asymptomatic positive antibody test, *E* enrolled at time of diagnosis, *M1* one month of recovery, *Neg* negative antibody test, *COVID-19* symptomatic patients. Data are means of ± SEM, and P < 0.05 is considered significant.

To further examine whether decreased serum sphingosine and dh-sphingosine levels in COVID-19 patients is linked to disease severity, we obtained longitudinal serum samples from symptomatic patients enrolled at the time of diagnosis (E) or after 1 month of recovery (M1) (Figs. 2A–H and 3A–J). The data showed that serum levels of sphingosine and dh-sphingosine of COVID-19 patients at the time of diagnosis or after 1 month of recovery were decreased at a comparable rate regardless of the gender, about a 15-fold reduction to that of asymptomatic donors (Fig. 2A–H). Also, reduced sphingosine and dh-sphingosine levels were detected at similar levels in COVID-19 patients who were treated as an inpatient, outpatient, or intensive care unit (ICU) who had < 5 pmol or < 1 pmol/5 × 10^−5^ L serum levels of sphingosine or dh-sphingosine, respectively (Fig. 4A–D). These data suggest that while reduced serologic sphingosine and dh-sphingosine levels are associated with symptomatic COVID-19, they do not appear to monitor the disease's severity.

### Increased sphingosine in asymptomatic donors is associated with elevated AC in the serum

To determine if serologic sphingosine/ceramide metabolism in COVID-19 patients and/or asymptomatic individuals regulated by AC, we measured secreted and active AC protein^39^ in the serum by Western blotting using PAGE (Fig. 4E) or slot blot apparatus using a commercially available anti-AC antibody (Fig. 4F,G). The data showed that out of 114 asymptomatic donors, 84 were positive for serum AC (73.7%), whereas only 2 out of 23 COVID-19 patients were positive for AC protein in their serum samples (8.6%), which were similar to the AC positive donors who were negative for the SARS-CoV-2 antibody (6 out of 23, 26%) (Fig. 4F). In addition, among longitudinal COVID-19 patient serum samples, we did not detect any upregulation of AC in E or outpatient samples after 1-month recovery or severity of COVID-19 disease, compared to asymptomatic donors (Fig. 4G). The original blots shown in Fig. 4E–G are included in Supplemental Fig. S1A–C. Thus, these data suggest that serologic AC protein levels are highly increased in most asymptomatic donors. However, most COVID-19 patients, regardless of disease severity, are negative for serum AC, which is also consistent with their decreased sphingosine and dh-sphingosine levels compared to asymptomatic donors.

## Discussion

COVID-19 pandemic continues to exert worldwide a health care emergency, and how some individuals stay asymptomatic despite SARS-CoV-2 infection remains largely unknown^2,3^. In this study, we compared the metabolic alterations concerning sphingolipids in SARS-CoV-2 antibody-positive donors who were asymptomatic versus symptomatic for COVID-19. Our data revealed that while most asymptomatic donors have a slight increase in their serum sphingosine, consistent with the presence of AC protein in their serum, symptomatic COVID-19 patients exhibited a robust decrease in their serum sphingosine levels, almost 15-fold reduction, compared to asymptomatic donors’s levels. In addition to the ROC analysis, our data demonstrated that most individuals (99.24%) whose serum sphingosine levels are between 0–8 pmol/5 × 10^−5^ L serum have developed COVID-19 symptoms compared to individuals whose serum sphingosine levels are between 8.4–23.25 pmol/5 × 10^−5^ L serum, supporting that serum sphingosine levels could be used as a sensitive and selective biomarker to predict positive COVD-19 symptomatic and asymptomatic patients. Although it is known that sphingosine and S1P levels are associated with increase in inflammation state locally, and that SARS-CoV-2 infections also induces inflammation, however, we didn’t observe a positive correlation between serum sphingosine, S1P, and SARS-CoV-2 infection.

Previous studies reported that the SARS-CoV-2 viral loads are usually similar in asymptomatic and symptomatic individuals, while asymptomatic individuals have higher immune cell counts, including T lymphocytes, B cells, or natural killer cells^40,41^. Although COVID-19 patients have decreased CD4^+^ and CD8^+^ T cells, increased IL-6 and IL-10 levels, associated with cytokine storm, are linked to disease severity^42,43^. However, the mechanisms behind why some SARS-CoV-2-infected individuals stay asymptomatic while others develop severe COVID-19 remain mostly unknown. It is also known that there is cross-reactivity among memory T cells against SARS-CoV-2 and common cold coronavirus epitopes, which may play a role in asymptomatic response in some individuals^9,44^. However, it will be essential to identify novel serological biomarkers for early identification of SARS-CoV-2-infected patients who would likely stay asymptomatic or develop COVID-19 symptoms.

It is also known that pro-inflammation and cytokine storm is associated with the severity of COVID-19 patients compared to asymptomatic individuals^45,46^. In addition to inflammatory cytokines, lipid metabolism alterations are also known to play a role in inflammation in response to bacterial and viral infections^47,48^. However, serologic alterations of biochemical metabolites, including sphingolipids, in SARS-CoV-2-infected symptomatic versus asymptomatic individuals have not been reported previously. To this end, our study provides a novel and selective serologic biomarker, reduction of sphingosine, which we believe would be clinically significant for the early detection of symptomatic versus asymptomatic individuals who are positive for SARS-CoV-2 antibody by providing early treatment decisions in the fight to stop the spread of the virus globally^49,50^.

One of the biochemical biomarkers, lactate dehydrogenase (LDH), was highly elevated in symptomatic patients with an increased mortality rate^51,52^. Interestingly, reduced sphingosine levels were not associated with disease severity in COVID-19 patients. These data also suggest that increased or sustained serum sphingosine levels, linked to higher serum AC protein, might prevent COVID-19 disease, while reduced sphingosine could result in enhanced inflammation and symptomatic response in some individuals. This is consistent with the effects of exogenous exposure of sphingosine on inhibiting the association between Spike protein and ACE receptors in vitro^36^, supporting the possible preventive roles of sphingosine in SARS-CoV-2 viral infection. There are reports which also showed that sphingosine plays a role in inhibiting pulmonary Pseudomonas and Staphylococcus aureus infections in cystic fibrosis^53–55^.

There are some limitations that need to be considered for the data presented in this work. For example, this study was performed retrospectively. It will be important to conduct similar studies in a prospective study with a larger cohort of donors to identify serologic sphingosine and AC measurements' efficiency in predicting future COVID-19 development in SARS-CoV-2-infected individuals. Also, since we don’t have symptoms onset information for the symptomatic COVID-19 patients, we don’t know exactly the delay between the onset of the symptoms and lipidomics measurements, which could influence sphingosine and acid ceramidase levels. Whether sphingosine and sphingolipid signaling play any roles and their mechanism of action in preventing viral infection or pro-inflammation/cytokine storm in individuals who are SARS-CoV-2 antibody-positive remains unknown and needs to be determined. Lastly, measurement of serum sphingolipid levels, particularly sphingosine, using mass spectrometry is relatively more expensive than simple ELISA-based assays. To this end, based on recent evidence that reduced serum S1P levels are highly associated with the severity of COVID-19 in patients^56^, it might be helpful to include S1P measurements also. These data are also consistent with recent studies which reported altered lipid metabolism associated with COVID-19 pathogenesis. For example, increased lipid accumulation was reported in SARS-CoV-2 infected cells and in the lungs of COVID-19 patients, suggesting a role for lipids in SARS-CoV-2 pathogenesis^57^. Also, low apo B/apo A1 ratio and low density lipoprotein-C (LDL-C) are observed to be predictive of renal deterioration in COVID-19 patients^58^, supporting the roles of alterations of lipid metabolism in the pathogenesis of COVID-19.

Overall, recent advances in lipidomics provide cost- and time-effective biomarker development and detection at a sizeable high-throughput scale to selectively identify symptomatic versus asymptomatic individuals who are positive for the SARS-CoV-2 antibodies. In conclusion, we report here a new and reliable serologic biomarker, sphingosine, whose reduced levels beyond 8.3 pmol/5 × 10^−5^ L serum is highly (99.24%) associated with symptomatic COVID-19 development. In contrast, SARS-CoV-2 antibody-positive individuals whose sphingosine levels are between 8.4 and > 23.25 pmol/5 × 10^−5^  serum will (99.2–99.9%) likely be asymptomatic. These results have vital biological implications to early detect symptomatic and asymptomatic individuals that could be important to make early treatment and/or social distancing decisions on a larger scale.

## Materials and methods

### Patients and serum samples

COVID-19 symptomatic patient samples were received from the MUSC COVID-19 Biorepository between May and July 2020. The samples were then heat inactivated at 56 °C in a water bath incubator for 1 h before further processing. The negatives and asymptomatic positives donor samples were received from the MUSC Center for Cellular Therapy (CCT)—that collects donor sample stocks from The Blood Connection (Charleston, SC). The Blood Connection is a blood center that collects donor samples from three US Southeast regions—South Carolina, North Carolina, and Georgia. Samples were collected from 46 counties in South Carolina, 56 counties in North Carolina, and 15 counties in Georgia. The negative control serum samples also known as true negatives, were collected from healthy donors by The Blood Connection at least more than a year before the COVID-19 pandemic started. See Tables 1, 2 and 3 for patient’s characteristics.

**Table 1:** Serum samples from healthy negative individuals, asymptomatic positives, and symptomatic COVID-19 patients.

	SARS-CoV-2 negative individuals (Neg)	SARS-CoV-2 positive asymptomatic patients (Pos)	Symptomatic patients (COVID-19)
Total number of serum samples	130	134	131
Sex (male:female:Unk)	50:80:0	56:70:8	44:48:39
Age range, years	17–85	16–76	20–94
Mean age, years (SD)	46.4 (15.6)	45.8 (16.1)	50.8 (17.6)
Race (W:B:A:Unk)	112:2:1:14	90:9:4:18	55:28:0:5
Hispanic or Latino	1	13	6

White (W), Caucasian/European; Black (B), African American/African; Asian (A); Unk (unknown); *SD* standard deviation.

**Table 2:** One-month longitudinal serum samples from symptomatic COVID-19 patients.

	Male		Female	
	Enroll (E)	Month 1 (M1)	Enroll (E)	Month 1 (M1)
Total number of serum samples	25	23	35	36
White	19	17	26	27
Black	4	4	8	8
Asian	0	0	1	1
Unknown	2	2	0	0

White, Caucasian/European; Black, African American/African.

**Table 3:** Symptomatic COVID-19 serum samples from outpatients, inpatients, and patients in intensive care units.

	Outpatient (Outpt)	Inpatient (Inpt)	Intensive Care Unit (ICU)
Total number of serum samples	46	27	23
Sex (male:female:Unk)	15:25:6	14:9:4	12:11:0
Mean age, years (SD)	43.3 (13.6)	51.9 (17.7)	59.4 (15.0)
Race(W:B:A:Unk)	34:4:0:2	13:7:0:0	9:14:0:0
Hispanic or Latino	2	2	0
Died	0	0	3
Mean time from symptoms to admission (day)	n/a	11.4	5.9
Ventilatory support	0	0	15
ARDS severity (range)	n/a	n/a	24–146

ARDS severity defined as PaO_2_/FiO_2_ (only reported in 5 subjects). White (W), Caucasian/European; Black (B), African American/African; Asian (A); Unk (unknown); *SD* standard deviation, *n/a* not available, *ARDS* acute respiratory distress syndrome, *PaO*_*2*_ oxygen in arterial blood, *FiO*_*2*_ oxygen in the inspired air.

### Inclusion and exclusion criteria

The negative and asymptomatic positive groups were determined based on IgG to SARS-CoV-2 spike protein Optical Density 490 (OD 490) values obtained from a microplate reader. OD 490 value less than 0.45 was defined as negatives, while OD 490 value of 0.45 or higher was defined as asymptomatic positives. Detail description of the SARS-CoV-2 spike protein assays used to classify negatives and asymptomatic positives for the same study cohort is reported in our recent publication^59^. However, in this study we only included values of OD 490 ≥ 1 as asymptomatic positive, while the asymptomatic positive OD 490 values between 0.45 and 1 were excluded since they were categorized as low positives. Both negative and asymptomatic positive individuals didn’t exhibit any COVID-19 related symptoms at the time of serum collection, while the COVID-19 individuals had common symptoms, including fever, cough, asthenia, shortness of breath, new loss of taste or smell, and/or sore throat with a positive PCR test at time of sample collection (Table 1). The 1-month longitudinal study included only positive symptomatic COVID-19 patients at time of their enrollment (Table 2). Finally, the outpatients, inpatients, and patients in intensive care units were all symptomatic and tested positive for COVID-19 virus (Table 3). The outpatients visited MUSC hospital for treatment but were not admitted, while inpatients were admitted to the university hospital for treatments. Patients in intensive care units were in critical conditions needing constant monitoring and medical supports like the ventilatory support.

### Ethics statement

All patient information’s were de-identified, and studies were performed under the approved protocols by the MUSC’s Institutional Review Board for Human Research (IRB, #: Pro00100487) and Institutional Biosafety Committee (IBC, #: IBC-2020-01064). Overall, all the studies reported here were performed based on the protocols approved by the institutional and/or licensing committee approving according to relevant guidelines and regulations. Studies involving specimens obtained from human participants were performed by approved protocols by the IRB at MUSC in accordance with relevant guidelines/regulations, and informed consent was obtained from all participants and/or their legal guardians by the COVID-19 Biorepository at MUSC.

### Mass spectrometry-based lipidomics

Separations for sphingolipids were performed by HPLC–MS/MS analyses at the MUSC Lipidomics Shared Resource as described^60^. The equipment consisted of a Thermo Scientific Vanquish uHPLC system coupled to a Thermo Scientific Quantum Access Max triple quadrupole mass spectrometer equipped with an ESI probe operating in the multiple reactions monitoring positive ion mode (MRM). Chromatographic separations were obtained under a gradient elution on a C8 column using a mobile phase with ammonium formate, formic acid in the water, and methanol, as previously described. Before analysis, samples undergo an ethyl acetate/isopropanol liquid–liquid extraction. Quantitative analyses of sphingolipids are based on eight-point calibration curves generated for each target analyte. The synthetic standards and a set of internal standards are spiked into an artificial matrix; they are then subjected to identical extraction procedure as the biological samples. These extracted standards are then analyzed with the samples by the HPLC–MS/MS. Peaks for the target analytes and internal standards are recorded and processed using the instrument’s software. Plotting the analyte/internal standard peak area ratios against analyte concentrations generates the sphingolipid specific calibration curves. Any sphingolipid for which no standards are available is quantitated using the calibration curve of its closest counterpart.

### Western blotting

AC protein, secreted to the serum in its active 13 kDa form (mature alpha-subunit), was detected using commercially available anti-AC antibody (BD Biosciences, #612302) by Western blotting after PAGE or slot-blot apparatus in serum samples obtained from SARS-CoV-2 antibody-negative or positive donors, who were asymptomatic or symptomatic.

### Statistical analysis

Data analysis represents Standard Error of the Mean (SEM) using Graph Pad Prism 9.0.0, and p < 0.05 were considered statistically significant for all comparisons. Normality and variance homogeneity assumptions were assessed. All continuous outcomes among 3 independent groups are compared using ANOVA tests. If a significant result is discovered, all possible pairwise comparisons are performed adjusting for multiple comparisons using Turkey’s method. For measures taken from the same subject at different time points, a paired t test is used to compare the mean difference at baseline and 1 month later. Unpaired *t* test is used to compare the mean difference of unrelated subjects. The ROC curve analysis was done using Graph Pad Prism 9.0.0, and the Wilson/Brown method was used to calculate the 95% confidence interval.

## Supplementary Information

Supplementary Figure S1.
