# Supplementary material for: Alterations of lipid metabolism provide serologic biomarkers for the detection of asymptomatic versus symptomatic COVID-19 patients
Source: Sci Rep. 2021 Jul 9;11:14232. doi: 10.1038/s41598-021-93857-7 (PMC8270895; doi:10.1038/s41598-021-93857-7)
Supplement: Supplementary file 1 — Supplementary Figure S1. [file 41598_2021_93857_MOESM1_ESM.pdf]

SUPPLEMENTARY MATERIALS

Alterations of lipid metabolism provide serologic biomarkers for the detection of asymptomatic versus symptomatic COVID-19 patients

Alhaji H. Janneh[1,2], Mohamed Faisal Kassir[1,2], Connor J. Dwyer[2,3], Paramita Chakraborty[2,3], Jason S. Pierce[1,2], Patrick A. Flume[2,4], Hong Li[2,5], Satish N. Nadig[2,3], Shikhar Mehrotra[2,3], and Besim Ogretmen[*1,2]

[1]Department of Biochemistry and Molecular Biology, [2]Hollings Cancer Center, [3]Department of Surgery, [4]Department of Medicine, [5]Department of Public Health Sciences, Medical University of South Carolina, 86 Jonathan Lucas Street, Charleston, SC 29425, USA

*Correspondence should be addressed to ogretmen@musc.edu

Supplemental Figure S1

A B C

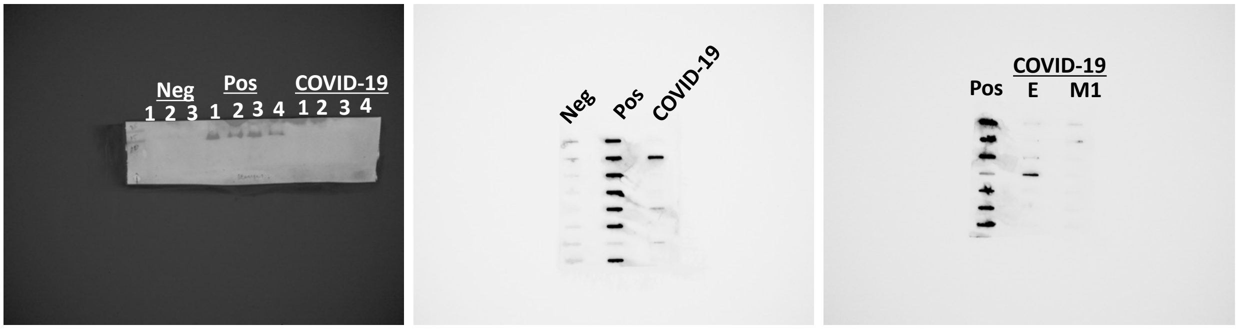

Western Blot-PAGE   Western-SLOT BLOT   Western-SLOT BLOT

Figure S1: **Original blot images**. **A**-**C**) Images show the full-length, uncropped Western blot images shown in main Figures 4E (A), F (B), and G (C) of the manuscript, respectively.
